# Auricular Ultrasonic Vagus Nerve Stimulation: Effectiveness of Blinding and the Occurrence of Adverse Effects in People with Tinnitus

**DOI:** 10.3390/brainsci16060586

**Published:** 2026-05-29

**Authors:** Poppy Hinton, Bas Labree, Marcus Kaiser, Mohamad A. Pourhoseingholi, Magdalena Sereda

**Affiliations:** 1 NIHR Nottingham Biomedical Research Centre, Nottingham NG1 5DU, UK; mzyph7@nottingham.ac.uk (P.H.); bas.labree1@nottingham.ac.uk (B.L.); amin.pourhoseingholi@nottingham.ac.uk (M.A.P.); 2 Hearing Sciences, Mental Health and Clinical Neurosciences, School of Medicine, University of Nottingham, Nottingham NG7 2RD, UK; 3 Precision Imaging, Mental Health and Clinical Neurosciences, School of Medicine, University of Nottingham, Nottingham NG7 2UH, UK; marcus.kaiser@nottingham.ac.uk; 4 Department of Neurosurgery, Ruijin Hospital, Shanghai Jiao Tong University School of Medicine, Shanghai 200025, China

**Keywords:** ultrasound stimulation, vagus nerve, blinding, adverse effects, tinnitus, neuromodulation

## Abstract

**Highlights:**

**What are the main findings?**

- Ultrasonic Vagus Nerve Stimulation (U-VNS) is highly blindable in people with tinnitus, using a sham device.
- U-VNS is associated with only mild and transient adverse effects.

**What are the implications of the main findings?**

- This effective blinding procedure will go on to inform the design of future clinical trials of U-VNS for tinnitus.
- The absence of serious adverse effects suggests these will not be a serious barrier to future trials of U-VNS in people with tinnitus.

**Abstract:**

**Background/Objectives**: Vagus Nerve Stimulation (VNS) has been suggested as a treatment for tinnitus, but its effect on the condition remains unclear. Ultrasonic Vagus Nerve Stimulation (U-VNS) involves non-invasive stimulation of the auricular branch of the vagus nerve, potentially providing an alternative to traditional VNS. To pre-emptively address some of the methodological challenges of future trials investigating U-VNS, a highly blindable sham device is needed. This study aimed to (1) investigate the effectiveness of blinding of a U-VNS device and (2) record any adverse effects, including any negative effects on tinnitus loudness, alongside their onset and duration. **Methods**: In this single-blind randomized controlled study, 20 volunteers with chronic tinnitus received two 29 min sessions of true U-VNS followed by sham U-VNS, or vice versa. Sessions were a week apart, and in a randomized order. The effectiveness of blinding and adverse effects, including changes in tinnitus loudness, were measured using self-report questionnaires. **Results**: James’ Blinding Index revealed that blinding was highly effective in both the real U-VNS condition, BI = 0.79, 95% CI (0.61–0.92), and the sham condition, BI = 0.76, 95% CI (0.60–0.89). Adverse effects were uncommon and mild, primarily consisting of sensations on the skin beneath the transducer. For most participants, tinnitus loudness either decreased or stayed the same in both conditions. **Conclusions**: A high level of blinding was achieved, suggesting that the ZenBud sham device may be suitable as an effective placebo control in future trials. Adverse effects were uncommon and mild. These findings will help inform the design of future clinical trials to evaluate the safety and efficacy of U-VNS.

## 1. Introduction

Tinnitus is defined as the perception of sound in the absence of an external auditory stimulus. It is commonly described as ringing, hissing or sizzling and is typically a subjective experience [1]. Subjective tinnitus affects approximately 14% of the general population and represents a major concern for over 120 million people worldwide, predominantly among individuals aged 65 years or older [2]. There are some physiological models which attempt to explain tinnitus. These include the neurophysiological model, the peripheral model and the central model [3,4]. These models propose that damage to the auditory system results in a change in central neural activity. Consequently, they explain why hearing loss is a risk factor for tinnitus, along with age, occupational noise exposure and otitis media, as these conditions can cause damage to inner ear structures [5]. Tinnitus can cause significant distress in affected individuals, and is associated with comorbidities such as insomnia, anxiety and depression [6]. Additionally, there appears to be a bidirectional link between stress and tinnitus. Tinnitus may lead to increased stress in an individual, which in turn maintains the percept [7].

Despite the proposed neurophysiological models of tinnitus previously discussed, the exact pathophysiology remains unclear. This is reflected in the limited effective treatment options for this condition [8]. There is currently no curative treatment for tinnitus, and current management strategies for chronic tinnitus focus on reducing its impact, rather than eliminating the tinnitus percept. Tinnitus management is challenging since the lack of good-quality research evidence results in difficulty making guidelines [9,10]. Current management options include sound therapy, which is usually used alongside patient education. This uses an auditory stimulus to provide a distraction from the percept, making it less audible. There is limited evidence to support its effectiveness, and although it is often used for short-term relief, it does not treat tinnitus [11]. Cognitive behavioral therapy (CBT) is currently the most evidence-based management approach [12], but this focuses on managing the emotional aspect of tinnitus, rather than reducing the percept [13]. The National Institute for Health and Care Excellence (NICE) recommends firstly supporting tinnitus patients and giving them information about the condition. At this stage they may also be referred to support groups or taught self-help strategies. For those who are still experiencing distress, a stepped psychological approach is recommended, which starts with digital CBT, followed by group interventions and then finally individual CBT [14].

Existing management options do not fully address patients’ needs, highlighting the need for additional interventions. Neuromodulation therapies, which target pathological neural activity in the brain that is associated with tinnitus, may be a potential future treatment option. One such neuromodulation therapy is Vagus Nerve Stimulation (VNS), which aims to disrupt pathological neural activity associated with tinnitus and has been proposed as a potential intervention to reduce the percept [13].

The vagus nerve is the tenth cranial nerve and originates in the medulla oblongata. With branches supplying the cranium, thorax and abdomen, it has the most extensive anatomical course and widest distribution of all the cranial nerves [15]. VNS promotes plasticity through the modulation of cortical neurons. This effect is mediated by the activation of the nucleus of the solitary tract, which subsequently engages the locus coeruleus and nucleus basalis, resulting in the release of neuromodulators which influence plasticity. Since tinnitus is associated with a dysregulation between excitatory and inhibitory neural activity which is associated with increased neuronal firing and reorganization of the auditory cortical map, VNS may represent a promising treatment for tinnitus. The proposed mechanism of action for stimulation of the vagus nerve is the disruption of this maladaptive neural activity via the activation of the nucleus of the solitary tract, leading to activation of the locus coeruleus and nucleus basalis, in turn leading to the release of neuromodulators that modulate the activity of auditory neurons, resulting in a reduction in tinnitus symptom severity. A systematic review found that, although most studies using VNS reported a small decrease in tinnitus distress after treatment, no strong conclusions can be drawn from the data due to small sample sizes and the risk of bias. Therefore, the effect of VNS on tinnitus remains unclear, highlighting the need for further research in this area [16].

Despite its potential therapeutic values, VNS usually involves an invasive procedure, which involves a surgically implanted electrode being wrapped around the vagus nerve [17]. This is associated with complications such as wound infections [18], and could also result in side effects such as throat pain, voice changes, dysphagia and cough [19]. To address this limitation, the transcutaneous delivery of electrical stimulation to the auricular branch of the vagus nerve, which can be accessed by the outer ear, has been suggested [20].

Typically, the vagus nerve is stimulated using electrical currents, but this creates a technical challenge: electrical signals diffuse as they are conducted through the skin. Ultrasound moves through tissue more focally, and so ultrasound stimulation may provide an alternative to electrical currents, allowing for more precise stimulation of the target site [21]. The safety profile of transcranial focused ultrasound stimulation is favorable, and the adverse effects seem to be rare [22].

**Figure 1:**
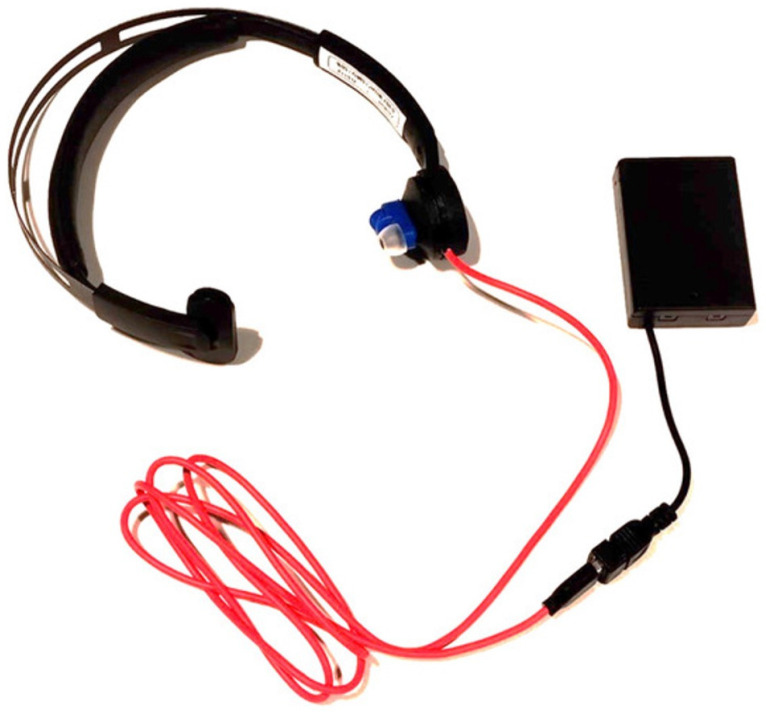
The ZenBud device.

Ultrasonic vagus nerve stimulation (U-VNS) can be delivered using the ZenBud device (Figure 1), which uses a transducer mounted in a headset to deliver focused ultrasound stimulation to the auricular branch of the vagus nerve. The device was developed by NeurGear Inc. and intended to relieve stress and anxiety. A study investigating the device for this purpose found that 92.9% of participants reported improvements in anxiety symptoms, and 83.1% said that they would continue to use the device if given the opportunity [23].

The ZenBud device might provide a way to deliver non-invasive U-VNS as a method to treat tinnitus, but appropriately statistically powered clinical trials would need to take place to determine its efficacy and safety. These trials would require an appropriate control condition. A sham version of the original ZenBud device could serve as a placebo control. Despite safety testing having taken place and the device receiving a CE mark, we still need to be aware of any adverse effects that stimulation might cause, specifically on people with tinnitus, so the participants of future trials are able to give their informed consent.

In a previous study, the ZenBud device was used to deliver U-VNS to 20 healthy participants, using the sham device as a control. Blinding, assessed using James’ Blinding Index, was shown to be highly effective and adverse effects were uncommon, mild and temporary. Adverse effects that were reported consisted mostly of sensations on the skin immediately beneath the transducer, described as tingling, burning or pain. These may have been associated with the pressure of the transducer on the skin rather than being an effect of the U-VNS itself [24].

This study aimed to replicate these findings in a sample of patients with tinnitus and to assess any tinnitus-related adverse effects, including changes in tinnitus loudness. This is necessary since these previous findings cannot be assumed to translate to the tinnitus patient population, and any immediate changes in tinnitus due to stimulation could have potentially led to unblinding. Before adequately powered clinical trials of the safety and efficacy of this technique can be conducted, certain methodological questions need to be addressed. This study sought to (1) investigate the effectiveness of blinding of a U-VNS device versus a sham control and (2) record any adverse effects that were experienced, along with their onset and duration.

## 2. Materials and Methods

This study received a favorable opinion from the Faculty of Medicine and Health Sciences Ethics Committee of the University of Nottingham (ethics reference number FMHS 183-0625). All data collection took place in a quiet laboratory at the NIHR Nottingham BRC facilities at Ropewalk House, Nottingham, UK, in the presence of either one or two of the authors.

### 2.1. Participants

A sample size calculation was carried out in accordance with recognized procedures appropriate for crossover designs [25]:

n = ((Z_{1−α/2} + Z_{1−β})^2 × 2 × (1 − ρ))/d^2

For a significance level (a) of 0.05 and to achieve 80% power (1 − β = 0.8), assuming a medium to large effect size (Cohen’s d = 0.7), based on adverse effects data from a previous study [24] and a moderate correlation between the conditions (ρ = 0.5), the required sample size for this study was determined to be 16. To account for attrition, 20 participants were recruited via emails to the participant database maintained by the Hearing theme of the NIHR Nottingham BRC. Potential participants were eligible for the study if they were aged 18 or over, had experienced constant idiopathic tinnitus for at least six months, were willing and able to give informed consent for participation in the study, were not currently taking any medication (except the contraceptive pill) and were able and willing to remove any piercings in the left ear. Participants were excluded if they had a current or past diagnosis of a neurological or psychological condition (including depression and anxiety), a current or past diagnosis of cardiac arrhythmia, had no tinnitus, had tinnitus that was objective, intermittent or had an onset of less than six months, had taken medication or recreational drugs that affect the nervous system in the past three months, were currently pregnant, were allergic to aquasonic gel or had taken part in a research study in the last three months that involved invasive procedures or inconvenience allowance.

### 2.2. Intervention and Comparator

The ZenBud device, developed by NeurGear Inc. (Rochester, NY, USA), was used to deliver U-VNS to participants in this study. The device is designed to deliver low-intensity focused ultrasound to the auricular branch of the vagus nerve through several layers of skin. The ultrasound stimulation is delivered at a pulse repetition frequency of 41 Hz, a center frequency of 5.3 MHz, a duty cycle of 50% and an average intensity of 1.03 MPa. The device turns off automatically after 29 min, and is designed to imitate a standard headset so it can be easily integrated into users’ routines [23]. The transducer usually sits on the right ear, but for this study a modified version of the device was used, where the transducer sat on the left ear. This aligns with previous VNS literature, which suggests that stimulation of the left vagus nerve is preferred.

A sham device, also developed by NeurGear Inc., was used to deliver the sham protocol. The device mimics the true ZenBud device and has an identical external appearance. Similarly to the active device, the transducer heats up slightly and a humming sound is used to indicate that it is on. Since this device is a sham version of the standard ZenBud, the (non-functional) transducer sat on the right ear.

### 2.3. Procedure

Participants attended two appointments, which were one week apart. All participants received both active U-VNS and sham stimulation, and the order in which these conditions took place was randomly assigned based on a random sequence, electronically generated by a research assistant who had no further involvement in the study. Ten participants experienced the sham condition in the first appointment and then active stimulation in the second, and the other ten received active U-VNS first followed by sham stimulation in the second session. Since the transducers of the active and sham devices sat on different ears, investigator blinding was not possible, so only participants were blinded.

In the first appointment, written consent was obtained and the eligibility of the participant was checked using a set of safety screening questions. Following this, participants completed the European School for Interdisciplinary Tinnitus Research Screening Questionnaire and the Tinnitus Functional Index [26,27]. Participants then filled out a Tinnitus Loudness VAS (range 0–10), which was filled out in both conditions. Next, participants underwent either active U-VNS or sham stimulation using the ZenBud device. This lasted for approximately 29 min, and during this time they sat in a quiet room the investigator. After this, in both sessions, participants filled out a second tinnitus loudness VAS, and then a questionnaire which assessed adverse effects, and asked participants if they thought they had received real or sham stimulation. Participants were unblinded verbally after the second session.

### 2.4. Data Analysis

To assess the effectiveness of blinding, participants filled out a questionnaire at the end of each session which asked “Do you believe you received real or sham stimulation”. They could choose from the options *real*, *sham* or *I don’t know*. These responses were used to calculate James’ Blinding Index [28].

$$BI=[1+P_{DK}+(1-P_{DK}\;)\;K_{D}\;]/2,where\;K_{D}=(P_{Do}-P_{De})/P_{De}$$

James’ Blinding Index is a statistical test of blinding effectiveness. It is expressed as number from 0 to 1, with 0 indicating complete unblinding and 1 indicating perfect blinding. For further details on this statistical test, see James et al. (1996) [28]. The questionnaire used to collect data on blinding and adverse effects can be found in the Supplementary Material. To determine the presence and severity of adverse effects, at the end of each session participants were asked to “Please indicate the extent to which, if any, you felt the following sensations by using the scale below”. They were able to choose from a list of adverse effects previously associated with tDCS: *Itching*, *Burning*, *Tingling*, *Pain*, *Warmth/heat*, *Headache*, *Fatigue*, *Nausea*, *Redness*, *Metallic taste*, *other* and the rating options *None*, *mild*, *Moderate* and *Severe*. Following this, participants were asked “How long did the sensations last?” with the options *It stopped quickly*, *It stopped around the middle of the session*, *It stopped around the end of the session*, and *It continued after the end of the session*.

## 3. Results

### 3.1. Demographic Information

Of the 20 participants, 14 (70%) were male and 6 (30%) were female. Participants were aged between 43 and 85 years (mean = 63.5 years, standard deviation 11.5). Tinnitus Functional Index scores ranged between 5 and 80.8 (mean = 36.9, standard deviation = 23.3).

### 3.2. Tinnitus Loudness

The loudness of participants’ tinnitus was assessed using a tinnitus loudness Visual Analog Scale (VAS), which they completed before and after stimulation in both conditions (see Table 1). In the active condition, eight participants reported that the loudness of their tinnitus decreased, with a reduction in scores ranging from 1 to 3 points (mean = 2, standard deviation = 0.76). Three people reported that their tinnitus increased in volume, with scores increasing by 1, 3 and 5 points. Nine people reported that there was no change in their tinnitus loudness. In the sham condition, ten participants noted that the volume of their tinnitus decreased, with the reductions in scores ranging from 1 to 5 (mean = 2.5, standard deviation = 1.27). Two participants reported an increase in loudness, with scores increasing by one point in both cases. Eight participants reported that the volume of their tinnitus stayed the same. Just one participant reported an increase in tinnitus loudness across both conditions.

### 3.3. Blinding

James’ Blinding Index (BI) was calculated to assess the effectiveness of blinding in this crossover trial. For the real intervention, the BI: 0.79, 95% CI: (0.61–0.92), and for the sham intervention, the BI: 0.76, 95% CI: (0.60–0.89), suggesting successful blinding with no systematic unblinding of participants, consistent across the two conditions. Results of the blinding questionnaire can be found in Table 2.

### 3.4. Adverse Effects

Adverse effects were uncommon, and those that were reported were mostly mild. Only one participant reported a moderate adverse effect, which was a ‘moderate warmth/heat’ in the active condition. No serious adverse effects were reported. The most reported adverse effects related to sensations on the skin immediately beneath the transducer, such as itching and pain. Many participants also reported experiencing mild redness, or warmth/heat in this area. The most common non-skin related adverse effects included headache and fatigue. Three participants experienced adverse effects not listed, which were described as ‘mild discomfort’ and ‘mild soreness’. One participant reported feeling a ‘fluttering in the ear following the removal of the device’. This only occurred for a few seconds and happened immediately after the removal of the device. The number of adverse effects reported was similar across both the active and sham conditions. Table 3 and Table 4 detail all adverse effects reported for both conditions. Due to the close similarity in the number of adverse effects reported, no further statistical analysis was conducted.

## 4. Discussion

This study suggests that U-VNS can be effectively blinded using a sham device and further suggests that adverse effects are uncommon, mild and transient.

Most participants reported that the loudness of their tinnitus either decreased or stayed the same, in both the active and sham condition. This finding is encouraging, because it suggests an increase in tinnitus loudness is not observed, and therefore will not be a barrier to the use of this device in future trials. Those who experienced an increase in loudness in one condition usually reported that their tinnitus either decreased or stayed the same in the other conditions, with only one participant reporting an increase in volume across both conditions. This, alongside the similarity of the changes across both conditions, suggests that it was potentially the humming sound the device emits that was altering the volume of participants’ percepts. Tinnitus can be reactive to sound, which can worsen the percept for some people and improve it for others. Additionally, for some people, the loudness of their tinnitus can increase in quiet environments [29]. The room that the participants were in when filling out their Tinnitus Loudness VAS was quiet, so the lack of background noise may have worsened the percept for them. While it is promising that no additional adverse effect was found in the form of increased tinnitus loudness, it is important to note that this study was not designed or powered to measure any treatment effect on tinnitus loudness. Since we cannot conclude that any changes in tinnitus loudness were the result of the U-VNS rather than the devices’ sound output, future trials may seek to remove this sound.

The level of blinding effectiveness achieved in this study was high, which indicates that participants were generally unable to distinguish between the active and sham devices. This is promising for future trials, since the ZenBud sham device can be relied upon as a highly blindable placebo control, with the caveat that this study was powered based on adverse effects data, rather than data relating to blinding. It should be noted that investigator blinding was not possible in this study, due to the devices sitting on different ears. The active device was modified to stimulate the left vagus nerve, to align with previous literature which suggests that stimulation of the right vagus nerve is associated with a higher risk of cardiac complications [30]. The possibility of expectancy bias, subtle cueing, and/or the influence of selective adverse event reporting cannot be discounted. Future studies should look to modify the sham device, so that investigator blinding can be applied. Though no carryover effect is apparent in the data, it is not possible to determine with certainty, based on the current findings, whether a one-week washout period is sufficient.

Very few adverse effects were reported, in comparison to other non-invasive neuromodulation techniques [13] and to our previous study in healthy volunteers [24]. Most were mild and related to sensations on the skin underneath the transducer such as pain, redness and warmth/heat. These may have been the result of the pressure of the transducer on the skin and the warmth produced by the transducer, rather than being an effect of the U-VNS itself. This is supported by the fact that the number and nature of the adverse effects were similar across both conditions. One participant noted experiencing a temporary ‘fluttering in the ear following the removal of the device’. This may have been caused by the sudden release of pressure from the transducer on the ear. U-VNS avoids adverse effects associated with surgically implanted VNS, such as voice hoarseness, increased cough and dyspnoea [31]. Moreover, U-VNS eliminates the risk of surgical complications such as infection at the surgical site and permanent failure of the recurrent laryngeal nerve [32]. A study conducted on healthy participants reported that ultrasound stimulation was associated with fewer side effects compared to electrical stimulation [33]. The low incidence of adverse effects associated with ultrasound stimulation suggests that it may represent a viable alternative to traditional VNS.

The sample size calculation was powered based on previous adverse effects data, rather than blinding data. The TFI scores at baseline ranged widely, from 5 to 80.8. This represents a considerable heterogeneity of tinnitus symptom severity in the sample. The majority of the participants (70%) were men, which is not necessarily representative of this patient population. In order to limit, as far as possible, the influence of confounding factors, the exclusion criteria did not permit any tinnitus patients to take part if they received any treatment affecting the nervous system. This potentially restricts the external validity of these findings. The adverse effects questionnaire was originally developed for use in tDCS studies. Whilst the format allowed participants to report any kind of adverse effects (and indeed some did, see Table 3 and Table 4), it may be a suboptimal tool for recording ultrasound-induced adverse effects specifically. All these factors may affect the generalizability of the results. Whilst this study addresses two methodological questions which must be addressed before adequately powered randomized trials of U-VNS for tinnitus can take place, it does not address all of them. In particular, the mechanism of action by which U-VNS is purported to stimulate the vagus nerve will need to be verified and work is currently ongoing to address this question.

To conclude, this study demonstrated a high level of blinding for the ZenBud sham device, supporting its use as a placebo control in future studies investigating the efficacy and safety of the device for conditions involving the vagus nerve. The low incidence of adverse effects is promising for future research, and the findings on adverse effects reported in this study should be communicated to potential future participants as part as the informed consent process of future trials.

## 5. Conclusions

The findings of this study suggest that U-VNS can be effectively blinded using a sham device and further suggests that adverse effects are uncommon, mild and transient. These results are in line with our previous study on healthy volunteers [24]. These findings will go on to inform the design of future clinical trials of U-VNS for tinnitus. 

## Figures and Tables

**Table 1:** Tinnitus loudness.

		Mean	Standard Deviation
Real U-VNS	Before	6.2	2.6
	After	5.8	3.0
Sham U-VNS	Before	6.15	2.4
	After	5	2.7

**Table 2:** Results of the blinding questionnaire.

	N	Real	Sham	I Don’t Know
Real U-VNS	20	5	2	13
Sham U-VNS	20	4	4	12

**Table 3:** Results of the adverse effects questionnaire after real U-VNS.

	None	Mild	Moderate	Severe
Itching	19	1	0	0
Burning	16	4	0	0
Pain	19	1	0	0
Tingling	18	2	0	0
Headache	17	3	0	0
Warmth/heat	13	6	1	0
Metallic taste	20	0	0	0
Fatigue	18	2	0	0
Nausea	20	0	0	0
Redness	16	4	0	0
Other *	18	2	0	0
Total	194	25	1	0

* Other adverse effects reported: Mild discomfort, mild fluttering in the ear immediately after removal of the device.

**Table 4:** Results of the adverse effects questionnaire after sham U-VNS.

	None	Mild	Moderate	Severe
Itching	18	2	0	0
Burning	19	1	0	0
Pain	16	4	0	0
Tingling	18	2	0	0
Headache	18	2	0	0
Warmth/heat	16	4	0	0
Metallic taste	19	1	0	0
Fatigue	18	2	0	0
Nausea	20	0	0	0
Redness	16	4	0	0
Other *	19	1	0	0
Total	197	23	0	0

* Other adverse effect reported: Mild soreness.

## Data Availability

All data for which consent to share has been obtained will be shared via the University of Nottingham archive under a CC-BY license.

## Supplementary Materials

The following supporting information can be downloaded at: https://www.mdpi.com/article/10.3390/brainsci16060586/s1, BLAST II: Blinding and acceptability of ultrasonic vagus nerve stimulation (U-VNS).

## Abbreviations

The following abbreviations are used in this manuscript:

VNS	Vagus Nerve Stimulation
U-VNS	Ultrasonic Vagus Nerve Stimulation
CBT	Cognitive Behavioral Therapy
NICE	National Institute for Health and Care Excellence
VAS	Visual Analog Scale
