# Supplementary material for: Auricular Ultrasonic Vagus Nerve Stimulation: Effectiveness of Blinding and the Occurrence of Adverse Effects in People with Tinnitus
Source: Brain Sci. 2026 May 29;16(6):586. doi: 10.3390/brainsci16060586 (PMC13297629; doi:10.3390/brainsci16060586)
Supplement: Supplementary file 1 [file brainsci-16-00586-s001.zip › brainsci-4300502-supplementary.pdf]

Faculty of Medicine & Health Sciences
School of Medicine
NIHR Nottingham Biomedical research Centre
Ropewalk House
113 The Ropewalk
Nottingham
NG1 5DU

BLAST II: Blinding and acceptability of ultrasonic vagus nerve stimulation (U-VNS)

Effectiveness of blinding and adverse effects questionnaire

Participant number: ______________________________ **Date:** ____/____/_________

This questionnaire is used to record what you felt during your U-VNS session.

1. **Do you believe that you received real or sham stimulation?**
   - ☐ Real
   - ☐ Sham
   - ☐ I don't know

2. **Why did you choose the above answer?**

____

____

____

University of Nottingham
UK | CHINA | MALAYSIA

Faculty of Medicine & Health Sciences
School of Medicine
NIHR Nottingham Biomedical research Centre
Ropewalk House
113 The Ropewalk
Nottingham
NG1 5DU

3. **Please indicate the extent to which (if any) you felt the following sensations by using the scale below.**

					Do you think this is U-VNS related?
**Itching:**	☐ None	☐ Mild	☐ Moderate	☐ Severe	Yes/No
**Burning:**	☐ None	☐ Mild	☐ Moderate	☐ Severe	Yes/No
**Pain:**	☐ None	☐ Mild	☐ Moderate	☐ Severe	Yes/No
**Tingling:**	☐ None	☐ Mild	☐ Moderate	☐ Severe	Yes/No
**Headache:**	☐ None	☐ Mild	☐ Moderate	☐ Severe	Yes/No
**Warmth/Heat:**	☐ None	☐ Mild	☐ Moderate	☐ Severe	Yes/No
**Metallic/Iron taste:**	☐ None	☐ Mild	☐ Moderate	☐ Severe	Yes/No
**Fatigue:**	☐ None	☐ Mild	☐ Moderate	☐ Severe	Yes/No
**Nausea:**	☐ None	☐ Mild	☐ Moderate	☐ Severe	Yes/No
**Redness:**	☐ None	☐ Mild	☐ Moderate	☐ Severe	Yes/No
**Other:**					
____________________	☐ None	☐ Mild	☐ Moderate	☐ Severe	Yes/No
____________________	☐ None	☐ Mild	☐ Moderate	☐ Severe	Yes/No

4. **If you reported sensation(s), when did it/they first begin?**
   - ☐ At the beginning of the session
   - ☐ Around the middle of the session
   - ☐ Towards the end of the session

5. **How long did the sensation(s) last?**
   - ☐ It stopped quickly
   - ☐ It stopped around the middle of the session
   - ☐ It stopped around the end of the session
   - ☐ It continued after the end of the session

Faculty of Medicine & Health Sciences
School of Medicine
NIHR Nottingham Biomedical research Centre
Ropewalk House
113 The Ropewalk
Nottingham
NG1 5DU

6. Where were these sensations located?

☐ Around the earpiece

☐ Other: ______________________________

7. Do you have any further comments regarding U-VNS sensations?

____

____

____
